# Unveiling the anti-inflammatory potential of olive leaf phenolic extracts in diabetes-related endothelial dysfunction

**DOI:** 10.3389/fendo.2025.1671932

**Published:** 2025-10-14

**Authors:** Ilaria Cappellacci, Nadia Di Pietrantonio, Davide Viola, Gloria Formoso, Małgorzata Elżbieta Zujko, Beatriz Martín-García, Ana M. Gomez-Caravaca, Vito Verardo, Assunta Pandolfi, Caterina Pipino

**Affiliations:** ^1^ Department of Medical, Oral and Biotechnological Sciences, Center for Advanced Studies and Technology (CAST), University G. d’Annunzio Chieti-Pescara, Chieti, Italy; ^2^ Department of Medicine and Aging Sciences, Center for Advanced Studies and Technology (CAST), University G. d’Annunzio Chieti-Pescara, Chieti, Italy; ^3^ Department of Food Biotechnology, Faculty of Health Science, Medical University of Białystok, Białystok, Poland; ^4^ Department of Analytical Chemistry, University of Granada, Granada, Spain; ^5^ Institute of Nutrition and Food Technology ‘José Mataix’, Biomedical Research Centre, University of Granada, Granada, Spain; ^6^ Department of Nutrition and Food Science, University of Granada, Granada, Spain

**Keywords:** endothelial inflammation, olive leaf polyphenols, diabetes mellitus, NF-κB signaling pathway, VCAM-1 expression, monocyte adhesion

## Abstract

**Introduction:**

Diabetes mellitus is a severe metabolic disorder strongly linked to vascular complications driven by endothelial dysfunction, chronic inflammation, and oxidative stress. Novel strategies to mitigate endothelial activation are urgently needed. In this context, phenolic compounds derived from olive leaves, a byproduct of olive oil production, have shown promising potential in counteracting diabetes- associated endothelial inflammation. This study investigates the potential anti-inflammatory effect of polyphenol-rich extracts derived from two olive leaves Spanish monocultivars, Picual and Changlot Real, in human umbilical vein endothelial cells from healthy pregnancies (C-HUVEC) and gestational diabetes (GD-HUVEC), which serve as a relevant in vitro model of hyperglycemia-induced endothelial dysfunction.

**Methods:**

Olive leaf extracts were characterized by HPLC-ESI-TOF-MS. C-HUVEC and GD-HUVEC were treated with the extracts, and pro-inflammatory markers expression (NF-kB p65, MCP-1, and VCAM-1), NF-kB p65 phosphorylation, and monocyte adhesion were assessed under basal and TNFα-stimulated conditions using RT-PCR, flow cytometry, and adhesion assays.

**Results:**

Both Picual and Changlot Real extracts showed no cytotoxicity at concentrations up to 50 mg/mL. Treatment with 10 mg/mL of both extracts significantly reduced NF-kB p65 and MCP-1 gene expression, as well as NF-kB p65 phosphorylation, particularly in GD-HUVEC. VCAM-1 protein expression and TNFα-induced monocyte adhesion were also significantly decreased following extract treatment. Notably, Changlot Real exhibited a broader anti-inflammatory effect across both cell types, while Picual exerted a more selective effect in GD-HUVEC.

**Discussion:**

These findings support the anti-inflammatory activity of olive leaf polyphenols and highlight the potential of Changlot Real and Picual extracts in mitigating endothelial dysfunction associated with diabetes. By modulating the NF-kB–VCAM-1 axis, these compounds may attenuate endothelial activation, warranting further investigation into their possible role in the prevention or mitigation of diabetes-related vascular complications.

## Introduction

Diabetes Mellitus is a major global health challenge primarily characterized by chronic hyperglycemia and associated with a significantly increased risk of cardiovascular disease (CVD). Individuals with diabetes face a significantly higher probability of vascular complications, categorized as either microvascular, such as diabetic nephropathy and retinopathy, or macrovascular, including coronary artery disease, stroke, and peripheral artery disease (1–4).

A fundamental contributing factor to these complications is endothelial dysfunction which represents an early and critical step in diabetes-associated vascular disease progression. The dysregulation of endothelial function arises from an intricated interplay of metabolic disturbances including hyperglycemia, dyslipidemia and insulin resistance, all of which foster vascular inflammation, oxidative stress, arterial stiffness, and impaired blood flow regulation (5, 6).

Chronic inflammation and oxidative stress play a key role in this pathological process by activating the transcription factor nuclear factor kappa B (NF-κB), which in turn promotes the expression of pro-inflammatory cytokines and adhesion molecules, such as Vascular Cell Adhesion Molecule-1 (VCAM-1), Intercellular Adhesion Molecule-1 (ICAM-1), E-selectin, Interleukin-6 (IL-6), and Monocyte Chemoattractant Protein-1 (MCP-1). These mediators enhance monocyte adhesion to the endothelium, thereby contributing to atherosclerosis development (7, 8).

Despite significant progress in diabetes control, lifestyle modifications, including diet and exercise, remain the first-line interventions for type 2 diabetes (4). However, while such interventions have proven advantages in managing blood glucose levels, the strategies available at the moment to prevent and manage diabetes-related vascular complications remain insufficient. This highlights the need for additional complementary therapeutic approaches that could help reduce the vascular burden of diabetes. In this context, bioactive polyphenols derived from natural sources, especially from olive tree (*Olea europaea L.*), have gained substantial attention due to their potent anti-inflammatory, antioxidant, and cardioprotective effects. Olive tree-derived compounds have shown promise in modulating the inflammatory and oxidative processes that drive endothelial dysfunction, suggesting their potential important role in diabetes management (9–11).

The olive tree (*Olea europaea L.*), a cornerstone of the Mediterranean diet, is rich in polyphenolic compounds that are well-documented for their beneficial effects on human health (12). While much research on olive products has focused on the health benefits of extra virgin olive oil (EVOO), olive leaves, typically discarded as agricultural byproducts, represent an untapped resource of powerful bioactive compounds (13–15). Olive leaf extract (OLE), rich in polyphenols such as oleuropein, hydroxytyrosol, luteolin, and apigenin, has demonstrated strong antioxidant, anti-inflammatory, cardioprotective, and neuroprotective properties. Among these compounds, oleuropein stands out for its ability to modulate oxidative stress, inhibit platelet aggregation, and improve glucose homeostasis by enhancing insulin sensitivity and glycemic control (16–20).

Preclinical studies have demonstrated that OLE supplementation may counteract oxidative damage induced by diabetes by ameliorating antioxidant enzyme activity and reducing levels of inflammatory markers. In diabetic animal models, OLE has been shown to improve glycemic control, preserve pancreatic islet integrity, and mitigate metabolic disturbances (11, 21–23). Furthermore, OLE’s neuroprotective effects may help alleviate cognitive impairment associated with diabetes reducing oxidative stress in brain tissue (24). These findings underscore OLE potential as an adjunctive therapy in diabetes management offering new perspectives for addressing the disease’s systemic complications.

This study investigates the potential of olive leaf polyphenols to mitigate endothelial inflammation associated with diabetes. It focuses on the effects of polyphenol-rich extracts from two Spanish olive tree monocultivars, Picual and Changlot Real, on human umbilical vein endothelial cells (HUVEC) derived from healthy pregnancies (C-HUVEC) and from pregnant women with gestational diabetes (GD-HUVEC). The GD-HUVEC model, characterized by a stable proinflammatory phenotype due to epigenetic alterations induced by hyperglycemia during pregnancy (25–27) offers a relevant *in vitro* system for studying diabetes-related vascular dysfunction. By examining how these specific phenolic extracts modulate endothelial activation and inflammatory pathways, the study aims to explore their possible role as natural agents that might contribute to managing vascular complications in diabetes.

## Methods

### Olive leaf samples

Olive leaves samples from cultivar Changlot Real and Picual were provided by “IFAPA, Centro Alameda del Obispo” in Córdoba, Spain (37°51′36.5″ N 4°47′53.7″W). Both samples were grown under the same agronomic and environmental conditions in the same olive orchards, and the leaves were collected in July.

### Extraction of phenolic compounds and their analysis by HPLC-ESI-TOF-MS

According to Talhaoui et al. (28), 0.5 g of dried leaf were extracted with methanol/water 80/20 v/v. The extracts were reconstituted with methanol/water 50/50 v/v and they were analyzed by HPLC-TOF-MS. The determination of targeted compounds was done using an Agilent 1200 HPLC system coupled to a QTOF Agilent 6520 B mass spectrometer using the column and chromatographic condition reported by Talhoui et al. (28).

### Clinical characteristics of cords donors and newborns

Umbilical cords were obtained from randomly recruited healthy Caucasian mothers (Control, C) and women affected by Gestational Diabetes (GD) in the third trimester of pregnancy and followed by ‘Diabetes and Pregnancy Clinic’ until delivery at the Hospital Santo Spirito of Pescara (Italy).

GD women were treated with diet only and women with pre‐gestational diabetes were excluded. All procedures agreed with the Declaration of Helsinki principles and with the ethical standards of the Institutional Committee on Human Experimentation. After approval of the protocol by the Institutional Review Board, signed informed consent was obtained from each participating subject. The characteristics of donors’ Control (n = 4) and GD (n = 4) women together with the newborns are described in **Table 1**. Women matched by age and body mass index (BMI) were divided into two groups, one normoglycemic/healthy controls (basal glycaemia < 5.1 mmol/L, oral glucose tolerance test (OGTT) 1 h < 10 mmol/L and OGTT 2 h < 8.5 mmol/L, n = 4) and the other diagnosed with gestational diabetes (basal glycaemia ≥ 5.1 mmol/L, OGTT 1 h ≥ 10 mmol/L, and OGTT 2 h ≥ 8.5 mmol/L, GD, n = 4) according to the criteria of the American Diabetes Association (29).

**Table 1:** Anthropometric and biochemical characteristics of control women, women with gestational diabetes and newborns.

Pregnant women	Control	GD	*p*
women (n)	4	4	
age (years)	35 ± 2	35 ± 6	0.9
OGTT (gestational week)	26 ± 2	26 ± 1	0.6
basal glycaemia (mmol/L)	4.4 ± 0.4	5.1 ± 0.2	0.01
1 h glycaemia (mmol/L)	5.8 ± 1	10.4 ± 0.9	0.001
2 h glycaemia (mmol/L)	4.4 ± 0.7	9 ± 1	0.001
height (cm)	162 ± 8	161 ± 6	0.8
pre-gestational weight (kg)	60 ± 7	67 ± 17	0.4
post-gestational weight (kg)	71 ± 8	76 ± 15	0.5
gestational weight gain (kg)	11 ± 3	9 ± 3	0.3
pre-gestational BMI (kg/m^2^)	23 ± 1	26 ± 5	0.3
post-gestational BMI (kg/m^2^)	27 ± 2	29 ± 4	0.4
SBP (mmHg)	112 ± 13	112 ± 5	1
DBP (mmHg)	72 ± 12	65 ± 4	0.3
GA at delivery	40 ± 0.5	38 ± 1	0.1

Newborns	Control	GD	*p*
Newborns (n)	4	4	
Sex (female/male)	3/1	2/2	
Birth Height (cm)	51 ± 1.5	50 ± 0.5	0.2
Birth Weight (kg)	3.4 ± 0.4	3 ± 0.2	0.2

Data are expressed as mean ± SD; GD, gestational diabetes; BMI, body mass index; OGTT, oral glucose tolerance test; SBP, systolic blood pressure; DBP, diastolic blood pressure; GA, gestational age.

### HUVEC isolation and culture

Primary human umbilical vein endothelial cells (HUVEC) were isolated from umbilical cords of newborns delivered between the 37th and 40th gestational weeks at the hospitals of Chieti and Pescara (Italy). Donors were randomly selected Caucasian mothers, either diagnosed with gestational diabetes (GD) or serving as healthy controls (C), following previously established protocols (7, 8).

Briefly, umbilical cords were collected immediately after delivery, and the veins were cannulated and enzymatically digested with 1 mg/mL collagenase 1A at 37 °C. The resulting HUVEC were isolated and cultured in a basal medium consisting of DMEM/M199 (1:1), supplemented with 1% L-glutamine, 1% penicillin/streptomycin, and 20% fetal bovine serum (FBS) (ThermoFisher; Waltham, MA, USA). After centrifugation at 1,200 rpm for 10 minutes, the cell pellet was resuspended in the same medium and plated onto 1.5% gelatin-coated culture flasks (Sigma-Aldrich, Germany).

Phenotypic characterization of HUVEC was confirmed by expression of endothelial markers such as von Willebrand factor, CD31, and CD34, along with inducible expression of adhesion molecules (ICAM-1, VCAM-1, and E-selectin) and cytokines (IL-6 and IL-8) upon pro-inflammatory stimulation (7, 8, 15, 30). Functional validation showed their ability to form capillary-like structures on Matrigel. Notably, GD-HUVEC formed less interconnected tubes with fewer segments, meshes, junctions, and nodes compared to controls, confirming impaired angiogenic capacity (30, 31).

Furthermore, GD-HUVEC showed a stable pro-inflammatory phenotype caused by epigenetic modifications acquired during *in vivo* hyperglycemia exposure (26). Accordingly, in this study GD-HUVEC demonstrated a significant upregulation of *NF-kB p65* gene expression and VCAM-1 protein expression compared to control cells (**Supplementary Figure 1**). Cells used for experiments were cultured between passages 3 and 5, with passage 5 never exceeded. For assays, HUVEC were maintained on 1.5% gelatin-coated plates in complete endothelial growth medium composed of low-glucose (1 g/L) DMEM and M199 (1:1), supplemented with 10 mg/mL heparin, 50 mg/mL endothelial cell growth factor (ECGF), 20% FBS, 1% penicillin/streptomycin, and 1% L-glutamine (all from Sigma-Aldrich, Germany).

**Figure 1:**
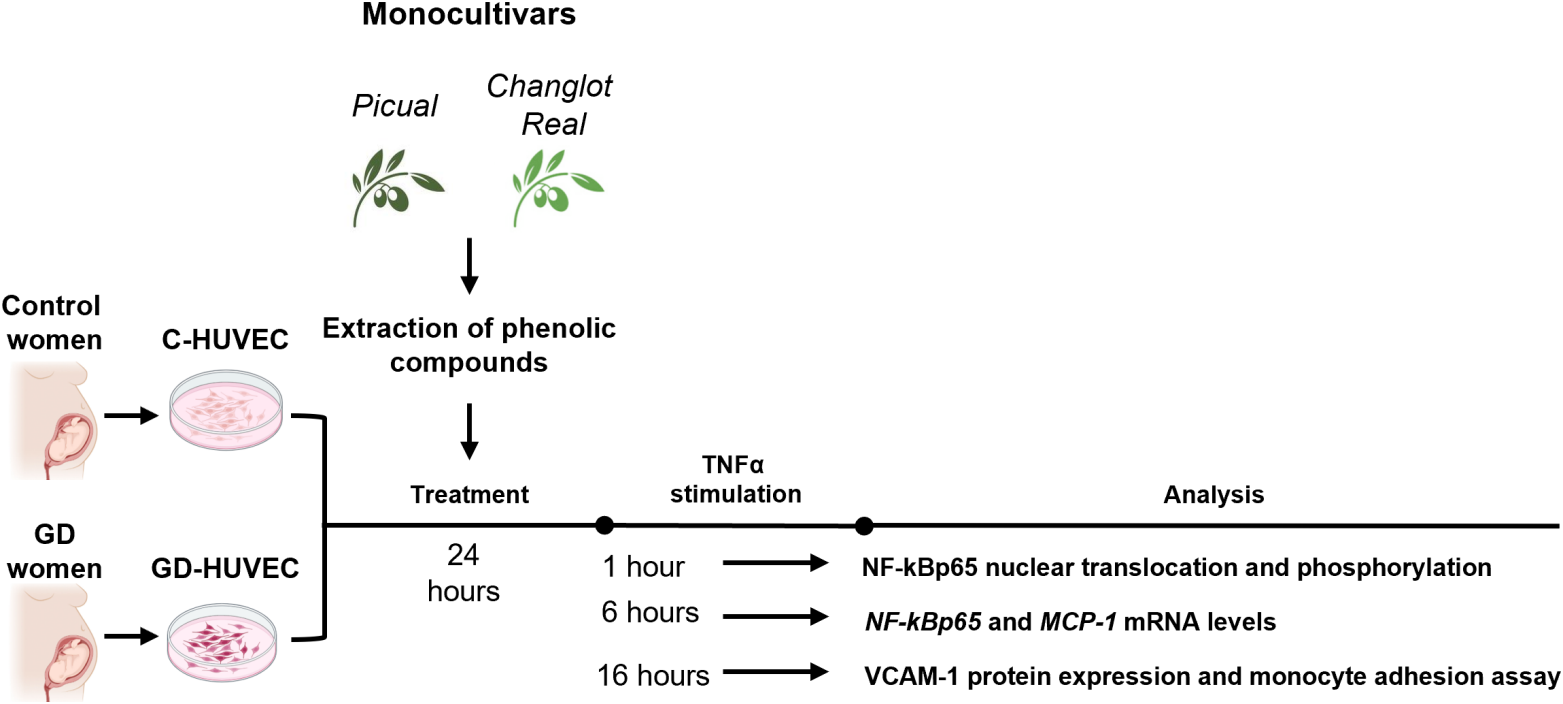
Experimental plan. Briefly, C- and GD-HUVEC were pre-treated with Changlot Real and Picual phenolic extracts for 24h (10 μg/mL) and then, cells were stimulated with TNFα (10 ng/mL) at different timing (1h, 6h and 24h) according to the experiment performed.

All experiments were performed in technical triplicates using HUVECs from at least three independent donors per group (control and GD, n = 3). TNFα exposure times were chosen to reflect the temporal dynamics of the NF-κB cascade: 1h to capture rapid p65 phosphorylation, 6h to monitor transcriptional activation, and 16h to assess late functional effects such as VCAM-1 surface expression and leukocyte adhesion (8, 26, 32) (**Figure 1**).

### MTT assay

Experiments were conducted on HUVEC isolated from control women and those diagnosed with gestational diabetes, following a previously established protocol (26). To determine the effect of polyphenol extract Picual and Changlot Real on C- and GD-HUVEC viability, the 3-(4,5-dimethylthiazolyl-2)-2, 5-diphenyltetrazolium bromide (MTT) assay (cat # M2128, Sigma‐Aldrich, Germany) was carried out. HUVEC were seeded in 96-well plates at a density of 5.600 cells/cm^2^. After 24 hours treatment with increasing concentration of extracts (0.1-100 μg/mL), cells were incubated for 3 hours at 37 °C with 5 mg/mL of MTT dissolved in PBS. Thereafter, MTT crystals were dissolved by DMSO addition (200 μL per well) and gentle shaking for 30 minutes. Finally, the absorbance of each sample was recorded at 540 nm using a microplate spectrophotometer system (Multimode Microplate Reader, BioTek Synergy H1, Agilent).

### RNA extraction and real-time quantitative PCR

The total RNA was isolated and extracted from HUVEC, treated with Picual and Changlot Real polyphenol extracts 10µg/mL, using the TRIzol reagent (Sigma-Aldrich, Germany) protocol. Quantification of recovered RNA was assessed using NanoDrop 2000 spectrophotometer (Thermo Scientific; Waltham, MA, USA). The High-Capacity cDNA Reverse Transcription Kit was employed to synthesize cDNA. The TaqMan Universal Master Mix II and TaqMan Gene Expression Assay probes for human NF-κB p65 (Hs01042014_m1), MCP-1 (Hs00234140_m1) and RPLP0 (Hs00420895_gH) were used according to the manufacturer’s instructions. All samples were analyzed in technical duplicate. The relative expression of the target genes was calculated using the 2^-ΔCt^ method using RPLP0 as housekeeping (Applied Biosystems QuantStudio 7 Pro Real-Time PCR System, Thermo Fisher Scientific).

### Immunofluorescence analysis

HUVEC were fixed with 3% formaldehyde for 20 min at RT. Formaldehyde was then removed and cells were washed with PBS for 5 min twice, and permeabilized with 0.1% Triton X-100 (Sigma-Aldrich, Germany) in PBS for 10 min. Cells were blocked with 1% bovine serum (cat # A4503, Sigma-Aldrich, Germany) in PBS for 1 h at RT. Subsequently, cells were incubated 1 hour with primary antibody NF-κB p65 (cat # C22B4, Cell Signaling Technology, 1:50). After washing with PBS, cells were incubated with Alexa Fluor 488-conjugated secondary antibody (cat #A-11034, Thermo Fisher Scientific, 1:50) and Phalloidin (cat #A12379, Thermo Fisher Scientific, Karlsruhe, Germany) at RT for 1 h. Nuclei were stained DAPI mounting medium (4′6′-diamidino-2-phenylindole; Thermo Fisher Scientific, Karlsruhe, Germany). Finally, cells were washed with PBS and mounted with Fluorescence Mounting Medium (cat # S3023, Agilent Dako, USA). For the acquisition of the immunofluorescence signals, slides were observed under a confocal microscope (Zeiss LSM-800; Carl Zeiss Meditec AG, Oberkochen, Germany).

### Flow cytometry analysis

Flow cytometry analysis was performed using BD FACS Canto II flow cytometer. Specifically, 1×10^4^ events for each sample were analyzed using FACSDiva v 6.1.3, IDEAS software (BD Biosciences). Cells were pretreated with polyphenolic extracts for 24 hours and, subsequently, incubated with or without TNFα (10 ng/mL) for 16 hours to assess VCAM-1 protein expression, and for 1 hour to evaluate NF-κB p65 and its phosphorylated form phospho-NF-κB p65 S536. To determine protein expression, cells were permeabilized using the Intrasure kit (cat # 641778, BD Biosciences, Sweden), processed, and incubated with primary antibodies: anti-VCAM-1 PE conjugate (cat # 12-1069-42, Invitrogen, 1:100), anti-NF-κB p65 (cat # C22B4, Cell Signaling Technology, 1:100), and anti-phospho-NF-κB p65 S536 (cat #3033, Cell Signaling Technology, 1:750) for 30 minutes at 4 °C. A subsequent incubation with Alexa Fluor 488-conjugated secondary antibody (cat #A-11034, Thermo Fisher Scientific, 1:100) was performed for 30 minutes at 4 °C to assess NF-κB p65 and phospho-NF-κB p65 expression. All results are expressed as the Mean Fluorescence Intensity (M.F.I.) Ratio, calculated by dividing the M.F.I. of positive events by the M.F.I. of negative events (M.F.I. of the secondary antibody).

### Monocyte adhesion assay

Control- and GD-HUVEC were pretreated for 24 hours with polyphenol extracts Picual and Changlot Real at 10 μg/mL concentration in six-well tissue culture plates, starved for 2 hours with 0.1% FBS, and treated with or without TNFα (10ng/mL for 16 h) as inflammatory stimulus. Briefly, 1×10^6^ U937 cells [European Collection of Authenticated Cell Cultures (ECACC)], grown in RPMI 1640, were co-cultured with HUVEC under rotating conditions at RT. After 20 min, non-adhering cells were removed with PBS, and monolayers were fixed with 1% paraformaldehyde. Images were obtained from high power fields taken at half-radius distance from the center of the wells using an inverted optical microscope PAULA cell imager (Leica, Wetzlar, Germany). The number of adherent cells in four individual experiments was determined with ImageJ software.

### Statistical analysis

All experimental data are expressed as mean ± Standard Deviation (SD). Each experiment was conducted in technical triplicates using at least 3 independent donors of C- and GD-HUVEC. The normal distribution of quantitative data was assessed by the Shapiro–Wilk test. One-way ANOVA test followed by Tukey’s test was used for multiple comparisons. Probability values were calculated considering a 95% compatibility interval. All analyses were performed with GraphPad Prism (version 9, GraphPad Software).

## Results

### Determination of phenolic compounds in olive leaf extracts

Phenolic content of the two studied samples is reported in **Table 2**. A total of 36 phenolic compounds from secoiridoids, flavonoids, simple phenols, oleosides and elenolic acids were identified and quantified by HPLC-ESI-TOF-MS in both olive leaf extracts. Changlot Real showed higher content of phenolic compounds than Picual. As expected, secoiridoids was the main phenolic class in both cultivar (65.7-79.5%), being oleuropein the first compounds accounting from more than 53.5% of the phenolic content. Flavonoids ranged from 6 to 12% of total phenolic compounds in Changlot Real and Picual, respectively. Simple phenols (tyrosol and hydroxytyrosol derivatives) represented less than 1.6% in both cultivars. Oleosides were from 10 to 15% and, finally, elenolic acids ranged from 3.5 to 6% of total phenolic compounds. These data are consistent with the previous ones of Talhoui et al. (28).

**Table 2:** Phenolic compounds in Changlot Real and Picual extracts quantified by HPLC-ESI-TOF-MS presented as percentage (%) and as concentration (mg/g).

N.	Name	Changlot Real (%)	Changlot Real (mg/g)	Picual (%)	Picual (mg/g)
1	Hydroxytyrosol-hexose isomer a	0,01	0.001 ± 0.0002	0,01	0.001 ± 0.0001
2	Oleoside	1,16	0.17 ± 0.2	0,82	0.07 ± 0.1
3	Hydroxytyrosol-hexose isomer b	0,66	0.10 ± 0.1	1,03	0.09 ± 0.05
4	Hydroxytyrosol	0,11	0.02 ± 0.001	0,36	0.03 ± 0.001
5	Secologanoside isomer a	7,96	1.17 ± 0.2	7,55	0.66 ± 0.1
6	Tyrosol glucoside	0,35	0.05 ± 0.002	0,07	0.01 ± 0.002
7	Caffeoyl glucoside	0,26	0.04 ± 0.003	0,06	0.01 ± 0.001
8	Tyrosol	0,04	0.01 ± 0.001	0,06	0.01 ± 0.001
9	Elenolic acid glucoside isomer a	0,10	0.01 ± 0.001	0,22	0.02 ± 0.001
10	Secologanoside isomer b	1,16	0.17 ± 0.1	6,93	0.60 ± 0.005
11	Elenolic acid glucoside isomer b	1,31	0.19 ± 0.2	2,59	0.23 ± 0.1
12	Oleuropein aglycon	2,55	0.37 ± 0.1	4,00	0.35 ± 0.2
13	Elenolic acid glucoside isomer c	1,96	0.29 ± 0.2	2,82	0.25 ± 0.1
14	Luteolin diglucoside	0,05	0.01 ± 0.002	0,16	0.01 ± 0.002
15	Elenolic acid glucoside isomer d	0,15	0.02 ± 0.001	0,38	0.03 ± 0.001
16	Demethyloleuropein	0,37	0.05 ± 0.002	1,66	0.14 ± 0.1
17	Hydroxyoleuropein isomer a	0,36	0.05 ± 0.002	1,08	0.09 ± 0.001
18	Rutin	0,43	0.06 ± 0.002	0,58	0.05 ± 0.002
19	Luteolin rutinoside	0,05	0.01 ± 0.001	0,18	0.02 ± 0.001
20	Luteolin glucoside isomer a	2,84	0.42 ± 0.2	4,90	0.43 ± 0.1
21	Verbascoside	0,04	0.01 ± 0.001	0,03	0.003 ± 0.001
22	Hydroxyoleuropein isomer b	0,27	0.04 ± 0.001	0,27	0.02 ± 0.001
23	Apigenin rutinoside	0,08	0.01 ± 0.001	0,13	0.01 ± 0.001
24	Oleuropein diglucoside isomer a	0,07	0.01 ± 0.001	0,10	0.01 ± 0.001
25	Apigenin-7-glucoside	0,59	0.09 ± 0.002	0,40	0.03 ± 0.001
26	Oleuropein diglucoside isomer b	0,07	0.01 ± 0.001	0,12	0.01 ± 0.001
27	Luteolin glucoside isomer b	1,34	0.20 ± 0.01	3,17	0.28 ± 0.02
28	Oleuropein diglucoside isomer c	0,29	0.04 ± 0.003	0,17	0.01 ± 0.001
29	Chrysoeriol-7-O-glucoside	0,45	0.07 ± 0.002	1,00	0.09 ± 0.003
30	Luteolin glucoside isomer c	0,24	0.04 ± 0.001	0,90	0.08 ± 0.001
31	Oleuropein isomer a	67,35	9.90 ± 0.13	52,55	4.57 ± 0.19
32	Oleuropein isomer b	1,63	0.24 ± 0.03	1,10	0.10 ± 0.01
33	Oleuropein/Oleuroside2	4,29	0.63 ± 0.04	2,76	0.24 ± 0.01
34	Ligstroside aglycone	0,29	0.04 ± 0.003	1,21	0.11 ± 0.01
35	Ligstroside	1,96	0.29 ± 0.03	0,66	0.06 ± 0.003
36	Luteolin	0,02	0.003 ± 0.001	0,16	0.01 ± 0.001

### Effects of phenolic extracts Picual and Changlot Real on cell viability

**Figure 2:**
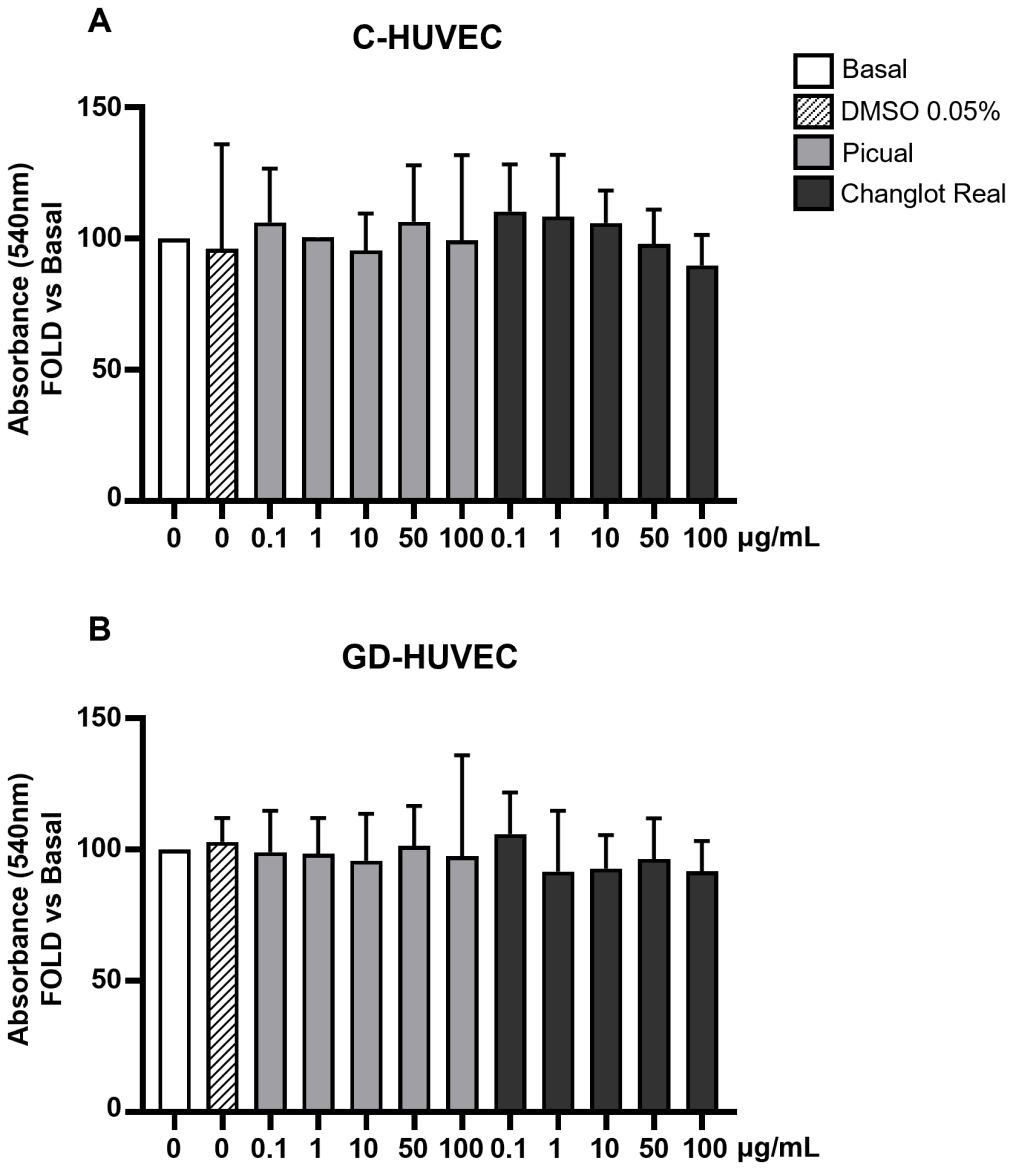
Changlot Real and Picual phenolic extracts treatment do not alter cell viability. Cell viability of C- **(A)** and GD-HUVEC **(B)** after 24 hours treatment with Changlot Real and Picual phenolic extracts at different concentrations (0.1–100 μg/mL) analyzed by MTT assay. Data are expressed as mean ± SD (n = 3).

To assess the possible cytotoxicity, polyphenol extracts derived from both Picual and Changlot Real olive leaves, two monocultivars widely spread throughout the Mediterranean basin, were tested using the MTT assay on both GD-HUVEC and C-HUVEC (**Figures 2A, B**). Cells were treated for 24 hours with four different concentrations of extracts (0.1, 1, 10, and 100 μg/mL) prepared in DMSO and added to the culture medium at a final DMSO concentration of 0.05%. Vehicle-matched controls were included in all experiments. The results showed no significant changes in cell viability at any concentration, indicating that both extracts are well tolerated and non-cytotoxic under the tested conditions.

### Effects of phenolic extracts Picual and Changlot Real on NF-κB p65 and MCP-1 mRNA Level

**Figure 3:**
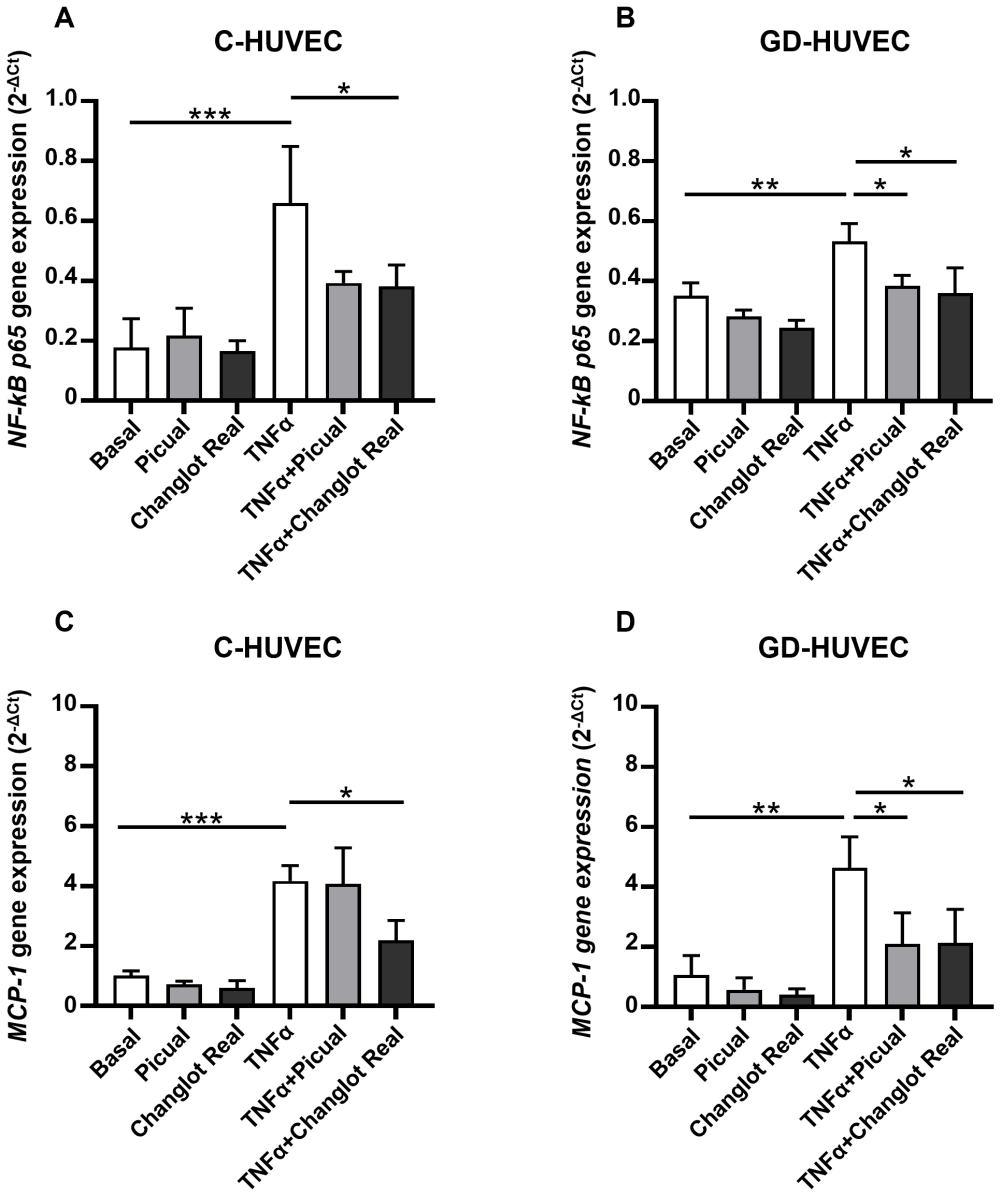
Changlot Real and Picual phenolic extracts treatment reduce *NF-κB p65* and *MCP-1* gene expression, with a more pronounced effect in GD-HUVEC. Relative mRNA expression of the inflammatory markers *NF-κB p65* **(A, B)** and *MCP-1* **(C, D)** in C- and GD-HUVEC following 24-hour treatment with Changlot Real and Picual phenolic extracts (10 μg/mL), with or without a 6-hour stimulation with TNFα (10 ng/mL) analyzed by RT-PCR. Data are expressed as mean ± SD (n = 3). Asterisks point out statistically significant differences between the selected conditions (*p<0.05; **p<0.01; ***p<0.001).

To evaluate and compare the possible anti-inflammatory effect of Picual and Changlot Real polyphenol extracts, the expression levels of the proinflammatory genes *NF-κB p65* and *MCP-1* were detected in HUVEC under different conditions (**Figure 3**). As expected, *NF-κB p65* and *MCP-1* basal expression was slightly higher in GD-HUVEC compared to controls. Following the selection of the optimal concentration by using a vehicle-matched controls (**Supplementary Figure 2**), cells were pre-treated with Picual and Changlot Real polyphenol extracts at 10 µg/mL. The results showed that both extracts led to a reduction in *NF-κB p65* and *MCP-1* gene expression, with a more pronounced effect in GD-HUVEC. In GD-HUVEC, a decrease, though not statistically significant, was also observed in the absence of a pro-inflammatory stimulus. (**Figures 3B-D**). Stimulation with TNFα (10 ng/mL) for 6 hours significantly upregulated *NF-κB p65* and *MCP-1* in both C-HUVEC and GD-HUVEC, indicating a robust inflammatory response. However, treatment with Changlot Real polyphenol extract effectively counteracted this effect, significantly reducing the expression of both genes in both cell types (**Figures 3A-D**). Similarly, Picual treatment also downregulated *NF-κB p65* and *MCP-1* expression; however, its effect was more pronounced and reached statistical significance in GD-HUVEC (**Figures 3B, D**). These findings indicate that both phenolic extracts may exert anti-inflammatory effects, with Changlot Real showing a broader impact across the tested cell conditions, whereas Picual appeared to have a marked effect primarily in GD-HUVEC.

### Effects of phenolic extracts Picual and Changlot Real on NF-κB p65 phosphorylation

**Figure 4:**
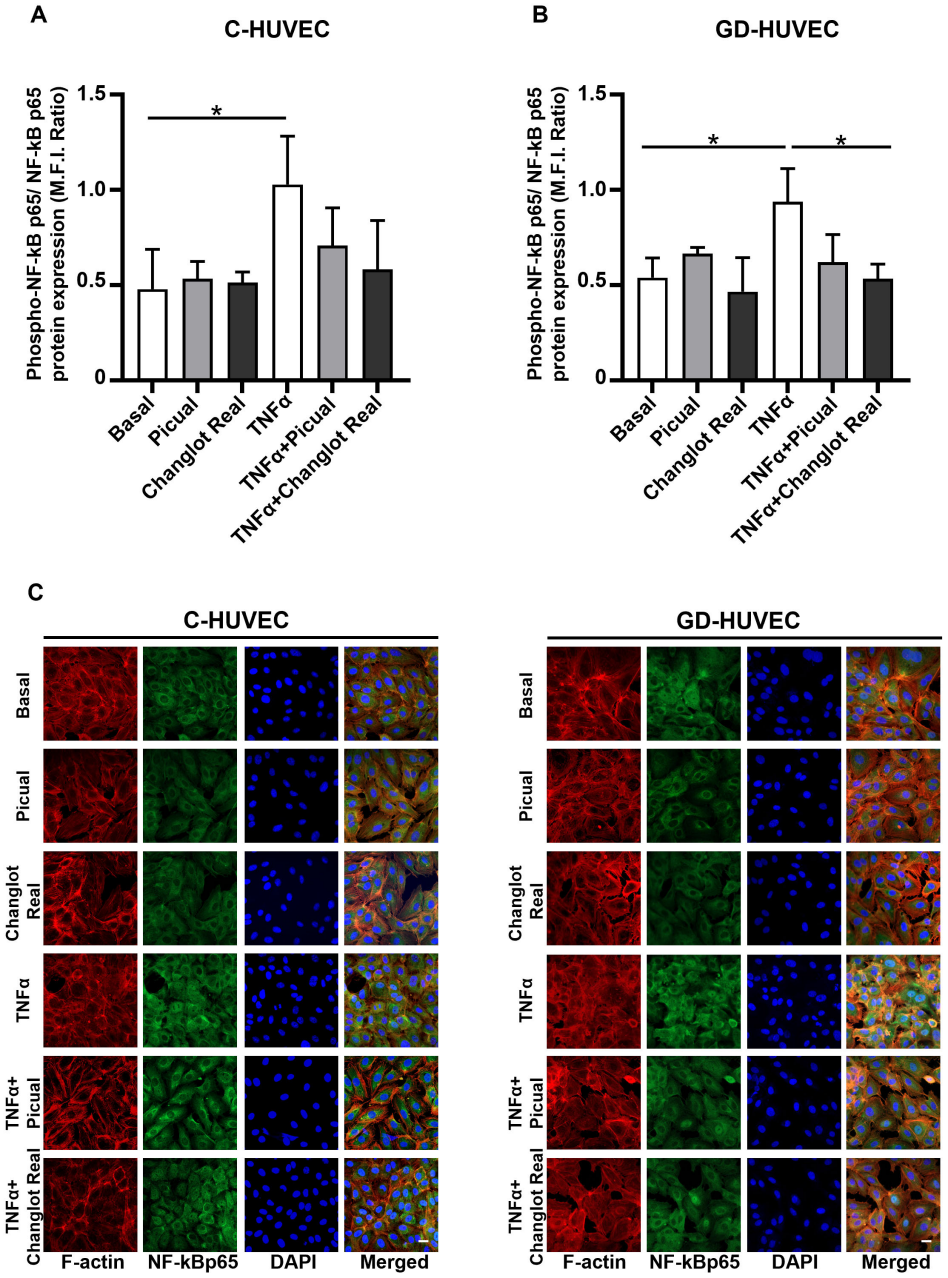
Changlot Real and Picual phenolic extract treatment reduces NF-κB p65 phosphorylation and nuclear translocation. Phosphorylated NF-κB p65 (Ser536) to total NF-κB p65 ratio in C- **(A)** and GD-HUVEC **(B)**. NF-κB p65 nuclear translocation representative figures in C- and GD-HUVEC **(C)**. Cells were treated for 24-hours with Changlot Real and Picual phenolic extracts (10 μg/mL), with or without 1-hour stimulation with TNFα (10 ng/mL). Histograms data are presented as mean ± SD (n = 3). Asterisks point out statistically significant differences between the selected conditions (*p<0.05). Scale bar 20 µm.

To further investigate the anti-inflammatory role of Picual and Changlot Real phenolic extracts, NF-κB p65 phosphorylation on Ser536 was evaluated in C- and GD-HUVEC (**Figure 4**). Under basal conditions, NF-κB p65 phosphorylation appeared modestly elevated in GD-HUVEC relative to control cells, though this difference was not statistically significant. Upon TNFα stimulation, a significant increase in NF-κB p65 phosphorylation was detected in both cell types. Pre-treatment with the Picual phenolic extract led to a marked reduction trend in NF-κB p65 phosphorylation in TNFα-stimulated C-HUVEC and GD-HUVEC. Similarly, pre-treatment with the Changlot Real polyphenol extract effectively counteracted TNFα-induced NF-κB p65 phosphorylation in both cell types, with a more pronounced and statistically significant effect in GD-HUVEC (**Figures 4A, B**). These findings align with NF-κB p65 nuclear translocation (**Figure 4C**) and previous data, suggesting that the anti-inflammatory effect of both phenolic extracts may be mediated through NF-κB pathway downregulation.

### Effects of phenolic extracts Picual and Changlot Real on VCAM-1 protein expression

**Figure 5:**
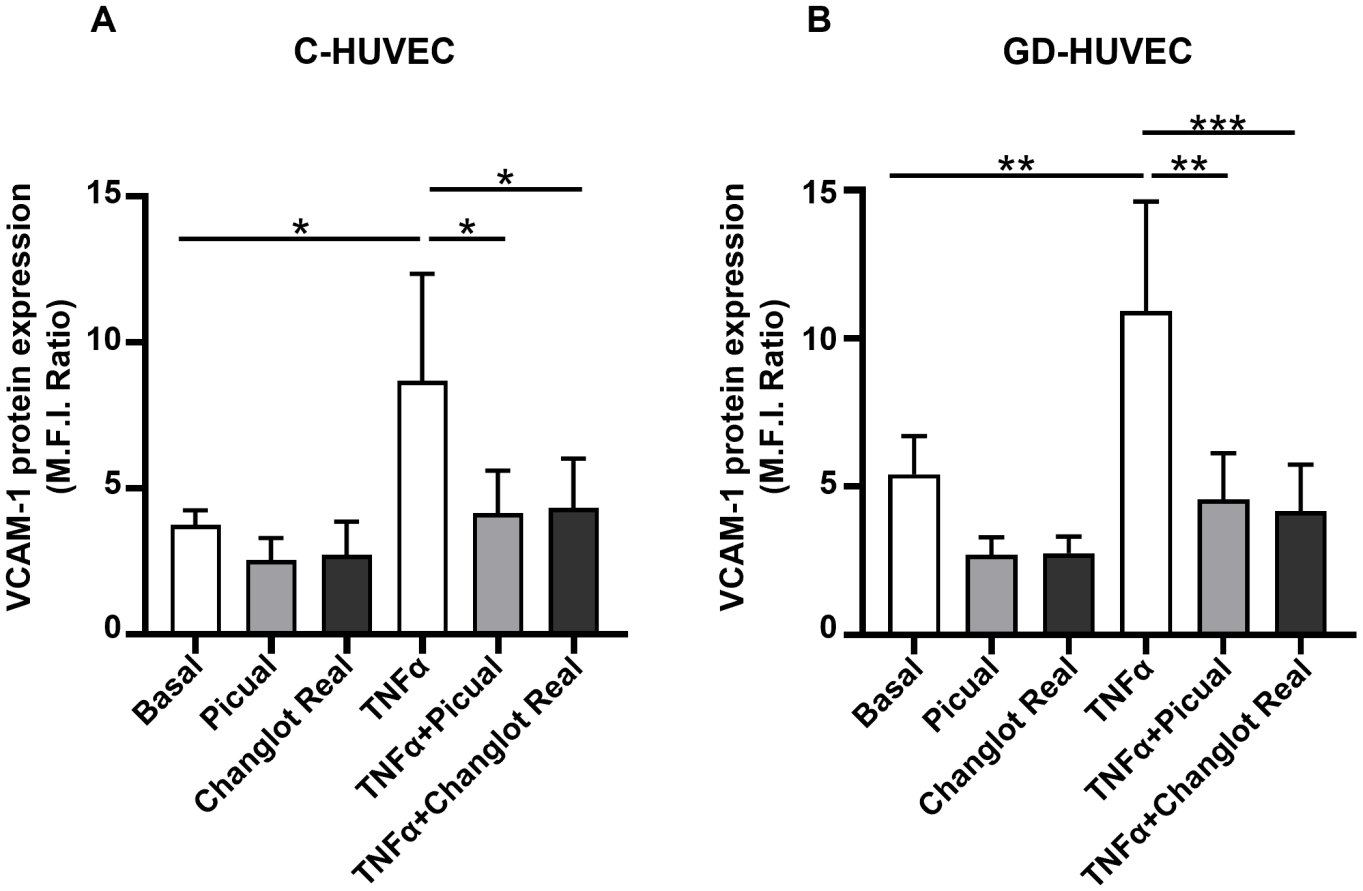
Changlot Real and Picual phenolic extracts treatment decreases VCAM-1 protein expression in TNFα-stimulated HUVEC. VCAM-1 protein expression in C- **(A)** and GD-HUVEC **(B)** following 24-hour treatment with Changlot Real and Picual phenolic extracts (10 μg/mL) with or without a 16-hour stimulation with TNFα (10ng/mL) analyzed by flow cytometry. Results are presented as mean ± SD (n=4). Asterisks point out statistically significant differences between the selected conditions (*p<0.05;**p<0.01;***p<0.001).

To further study the NF-κB p65 mediated pathway, VCAM-1 protein expression, a main downstream molecule that strongly modulates endothelial inflammation, was investigated in C- and GD-HUVEC treated with Picual and Changlot Real phenolic extracts with or without TNFα stimulation (**Figure 5**). GD-HUVEC exhibited a significantly increased basal VCAM-1 protein expression compared to control cells confirming their pro-inflammatory phenotype. Interestingly, treatment with Picual and Changlot Real extracts resulted in reduced VCAM-1 expression in GD-HUVEC; however, this reduction did not reach statistical significance. As expected, TNFα stimulation significantly increased VCAM-1 expression in both cell types, with a more pronounced effect in GD-HUVEC. Furthermore, the pre-treatment with both Picual and Changlot Real phenolic extracts caused a significant decrease in VCAM-1 expression induced by TNFα in both C- and GD-HUVEC, mostly in the latter one. These findings provide additional evidence of the possible capacity of Picual and Changlot Real extracts in modulating endothelial inflammation.

### Effects of phenolic extracts Picual and Changlot Real on U937 monocyte–HUVEC interaction

**Figure 6:**
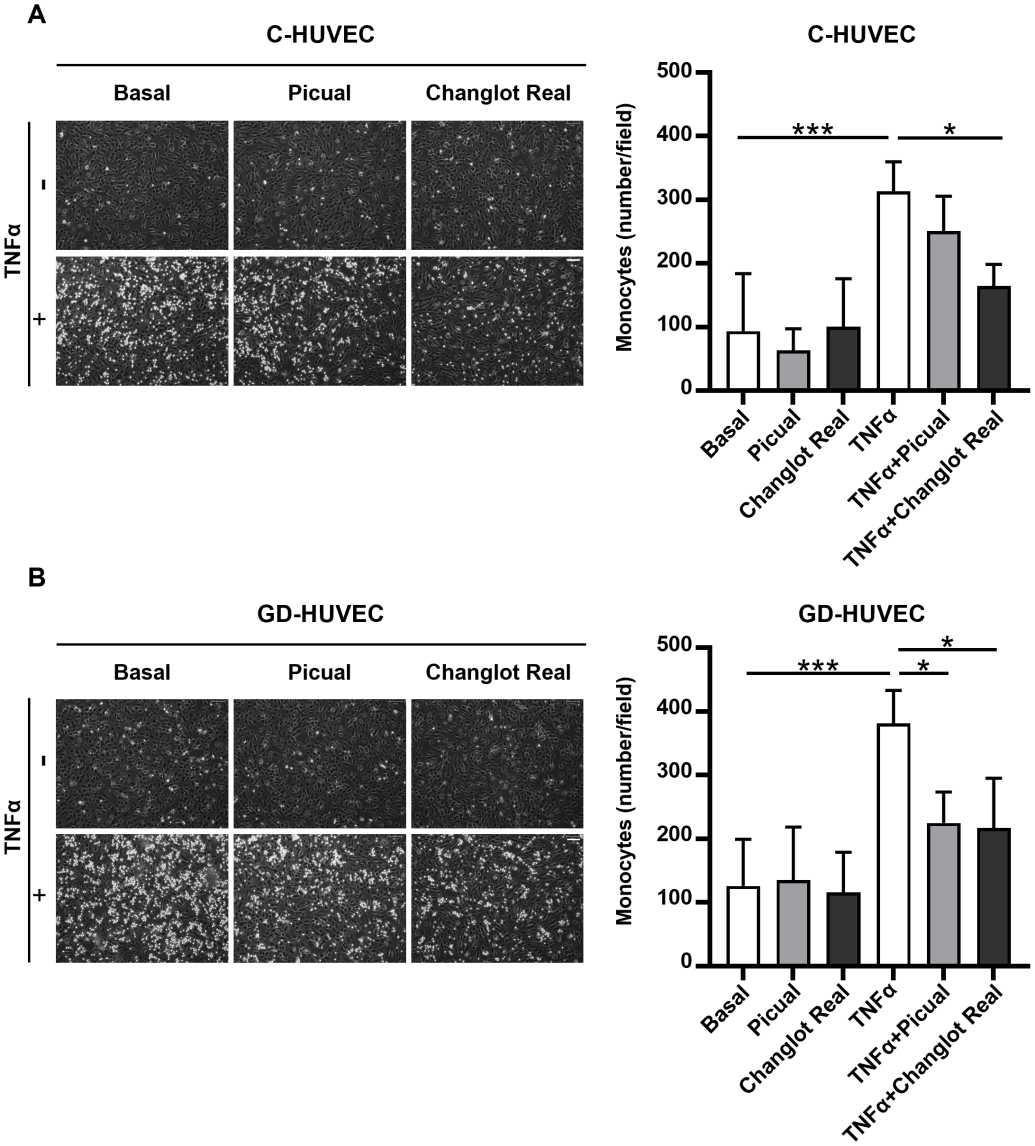
Changlot Real and Picual phenolic extracts treatment decreases monocytes adhesion in both cell types, with a more pronounced effect on Changlot Real pre-treated GD-HUVEC stimulated with TNFα. HUVEC–monocyte adhesion assay and relative quantification of adherent monocytes to C- **(A)** and GD-HUVEC **(B)** after 24-hour treatment with Changlot Real and Picual phenolic extracts (10 μg/mL), with or without 16-hour stimulation with TNFα (10 ng/mL). Data are expressed as mean ± SD (n = 4). Asterisks point out statistically significant differences between the selected conditions (*p<0.05; ***p<0.001). Scale bar 100 µm.

To assess the *in vitro* potential anti-inflammatory role of the phenolic extracts Picual and Changlot Real in a functionally relevant context, a monocyte adhesion assay was performed. This assay closely mimics the early stages of vascular inflammation observed in pathological conditions such as atherosclerosis. As shown in **Figure 6**, pre-treatment with the Changlot Real phenolic extract for 24 hours significantly reduced TNFα-stimulated monocyte adhesion to both C-HUVEC and GD-HUVEC, strongly supporting its anti-inflammatory effect on the endothelium. Pre-treatment with the Picual phenolic extract for 24 hours led to a decrease in TNFα-stimulated monocyte adhesion in C-HUVEC, although this reduction was only statistically significant in GD-HUVEC, supporting its selective effect in the diabetic-like endothelial model.

## Discussion

This study demonstrates the anti-inflammatory potential of phenolic extracts derived from *Picual* and *Changlot Real* monocultivar olive leaves, focusing on their effects on an *in vitro* model of endothelial cells derived from umbilical cords of neonates born to mothers with gestational diabetes and therefore exposed to hyperglycemia *in vivo* (26). The extracts were thoroughly characterized by HPLC-ESI-TOF-MS, revealing a rich and various phenolic composition. In particular, Changlot Real exhibited a higher total phenolic content than Picual, particularly in secoiridoids, known for their strong bioactivity. These results are consistent with previous reports, such as Talhoui and collaborators, confirming the prevalence of oleuropein as the major constituent (28).

The gestational diabetes-derived endothelial cell (GD-HUVEC) model represents a valuable *in vitro* system for studying the effects of chronic hyperglycemia on endothelial function. Having developed in a hyperglycemic intrauterine environment, these cells exhibit molecular and functional alterations that mirror diabetes-associated vascular dysfunction, including changes driven by epigenetic modifications (7, 26, 33). This makes GD-HUVEC a relevant model to investigate mechanisms underlying endothelial impairment, such as inflammation resulting from prolonged high glucose exposure. Consistent with our previous findings (26), we confirmed baseline differences between GD-HUVECs and control endothelial cells (C-HUVEC) in both NF-κB p65 gene expression and VCAM-1 protein levels, key regulators of endothelial inflammatory pathways.

Our data indicate that both Picual and Changlot extracts are non-toxic to endothelial cells across a wide range of concentrations (0.1–50 μg/mL). The cell viability of both control (C-HUVEC) and gestational diabetes-derived endothelial cells (GD-HUVEC) remained stable upon treatment, demonstrating that these extracts do not compromise cell integrity. This finding is crucial for potential therapeutic applications, as it confirms that the tested polyphenols can be used at these concentrations without adverse effects on endothelial cell viability.

At the molecular level, olive leaf extracts anti-inflammatory effects were evidenced by a consistent downregulation of key inflammatory markers. Specifically, pre-treatment with both Picual and Changlot Real significantly reduced *NF-κB p65* and *MCP-1* gene expression in both basal and TNFα-stimulated conditions. Interestingly, while Changlot Real showed a broader inhibitory effect across both control and GD-HUVEC, Picual exerted a more pronounced action exclusively in GD-HUVEC, which we have previously shown to exhibit an inflammatory phenotype due to epigenetic alterations induced by the diabetic environment (26). This suggests potential differences in the bioactive compound profiles or bioavailability between the two monocultivars, possibly linked to the higher phenolic content in Changlot Real.

Interestingly, both extracts attenuated NF-κB p65 phosphorylation at Ser536, a key activation step in the NF-κB signaling cascade, especially under TNFα stimulation. This inhibitory effect was more significant in GD-HUVEC, reinforcing the notion that these cells are particularly responsive to polyphenol treatment and that the extracts may be especially effective in pathological conditions associated with endothelial dysfunction.

Downstream NF-κB, VCAM-1, a critical adhesion molecule involved in monocyte recruitment during vascular inflammation, was also downregulated following treatment with both extracts. Again, Changlot Real and Picual reduced VCAM-1 expression more effectively in GD-HUVEC, even under basal conditions, indicating a preventive anti-inflammatory effect beyond the response to external stimuli. Similar results have been recently reported using olive leaf extract and its phenolics, oleuropein and hydroxytyrosol, to suppress LPS-induced VCAM-1 expression and monocyte adhesion in human coronary and umbilical endothelial cells (34, 35).

Functionally, this molecular modulation translated into reduced monocyte adhesion to the endothelium, an early crucial step in atherogenesis. Changlot Real significantly decreased TNFα-induced monocyte adhesion in both control and GD-HUVEC, while Picual exerted a comparable effect exclusively in GD-HUVEC. Collectively, these results confirm the anti-inflammatory activity of both extracts and suggest a potential disease-modifying effect under diabetic-like conditions, characterized by sustained oxidative stress and low-grade inflammation.

Taking together, these data indicate that Picual and Changlot Real olive leaf extracts exert their anti-inflammatory effects via the NF-κB pathway, reducing both transcriptional and post-translational activation steps, and ultimately limiting monocyte recruitment. The stronger and broader effect observed with Changlot Real may be attributed to its higher content of phenolic compounds, particularly secoiridoids, which are known to modulate oxidative and inflammatory pathways. Supporting this, studies on extra virgin olive oil and related supplements consistently report dose-dependent reductions in inflammatory cytokines and soluble adhesion molecules in humans, reinforcing the relevance of our *in vitro* findings (36). Further corroborating these results, our recent work demonstrated that oleanolic acid, a bioactive triterpenoid abundant in olive leaves and oil, lowers expression of key inflammatory adhesion molecules (VCAM1, ICAM1, SELE), reduces monocyte adhesion, and restores angiogenic function and migration in endothelial cells derived from gestational diabetic pregnancies, highlighting its potential to counteract hyperglycemia-induced endothelial dysfunction via epigenetic mechanisms (15). In addition, our *in vitro* findings align with previous *in vivo* studies demonstrating the beneficial effects of olive leaf extract (OLE) in ameliorating systemic inflammation and vascular dysfunction. For instance, OLE supplementation in aged rats improved endothelial function and metabolic parameters, including reductions in pro-inflammatory markers such as TNF-α and IL-6, together with enhanced antioxidant gene expression, highlighting its protective role against age-related vascular decline (15, 37, 38). Moreover, in a model of high-fat diet-induced obesity, OLE not only improved glycemic control and lipid profiles but also reduced systemic and adipose tissue inflammation, restored gut microbiota composition, and reversed endothelial dysfunction, indicating its multifaceted therapeutic potential in cardiometabolic diseases (15, 37, 38). These *in vivo* data strongly support our current observations and further underscore the translational relevance of OLE, particularly in pathological settings characterized by chronic inflammation and endothelial impairment. In addition, recognizing the importance of pharmacokinetics, bioavailability, and safety for therapeutic applications, previous toxicological studies have confirmed the safety of Olea europaea leaf extracts (39). Investigations into the bioavailability and metabolism of key polyphenols such as oleuropein and hydroxytyrosol provide evidence of their systemic activity in humans (40–42). Clinical trials have also reported improved insulin sensitivity following OLE supplementation (43), and herb-drug interaction studies suggest its safe use alongside antihypertensive medications (44).

In summary, our pilot *in vitro* study indicates that Picual and Changlot Real olive leaf polyphenols may modulate the NF-κB–VCAM-1 axis and reduce endothelial inflammation and monocyte adhesion. The observed stronger effect of Changlot Real suggests that monocultivar extracts with higher phenolic content could have potential relevance for further investigation in the prevention or mitigation of diabetic vascular complications.

The results of this study align with previous findings and suggest that monocultivar olive leaf polyphenols, particularly those from Changlot Real, may offer a promising natural and non-toxic approach to reducing endothelial inflammation, especially in the context of diabetes-related vascular complications. Nonetheless, a notable limitation of our work is the inability to evaluate the potential influence of sex on offspring outcomes, since several studies have suggested that sex differences can affect metabolic responses and long-term health during gestational diabetes (45–47). This aspect warrants further investigation. It should also be considered that our evidence is currently limited to *in vitro* observations, and that detailed phytochemical profiling of the individual bioactive constituents was beyond the scope of this study. Future research will focus on comprehensive chemical characterization and dose–response analyses to better define the active constituents and elucidate their mechanisms of action. Additionally, further investigations involving larger sample sizes, *in vivo* models, clinical trials, and appropriate positive pharmacological controls will be essential to confirm these preliminary findings and clarify the molecular pathways involved.

## Data Availability

The original contributions presented in the study are included in the article/**Supplementary Material**. Further inquiries can be directed to the corresponding author.
