# Supplementary material for: Unveiling the anti-inflammatory potential of olive leaf phenolic extracts in diabetes-related endothelial dysfunction
Source: Front Endocrinol (Lausanne). 2025 Oct 14;16:1671932. doi: 10.3389/fendo.2025.1671932 (PMC12558824; doi:10.3389/fendo.2025.1671932)
Supplement: Supplementary file 1 [file DataSheet1.docx]

**Supplementary Figure 1.** Basal differences between C- and GD-HUVEC. Relative mRNA expression of *NF-κB p65* (A) and *MCP-1* (B), NF-κB p65 phosphorylation (C), VCAM-1 protein levels (D) and monocytes adhesion (E) in C- and GD-HUVEC at basal conditions. Results are expressed as the mean ± SD (n≥3). Asterisks point out statistically significant differences between the selected conditions (*p<0.05).

**
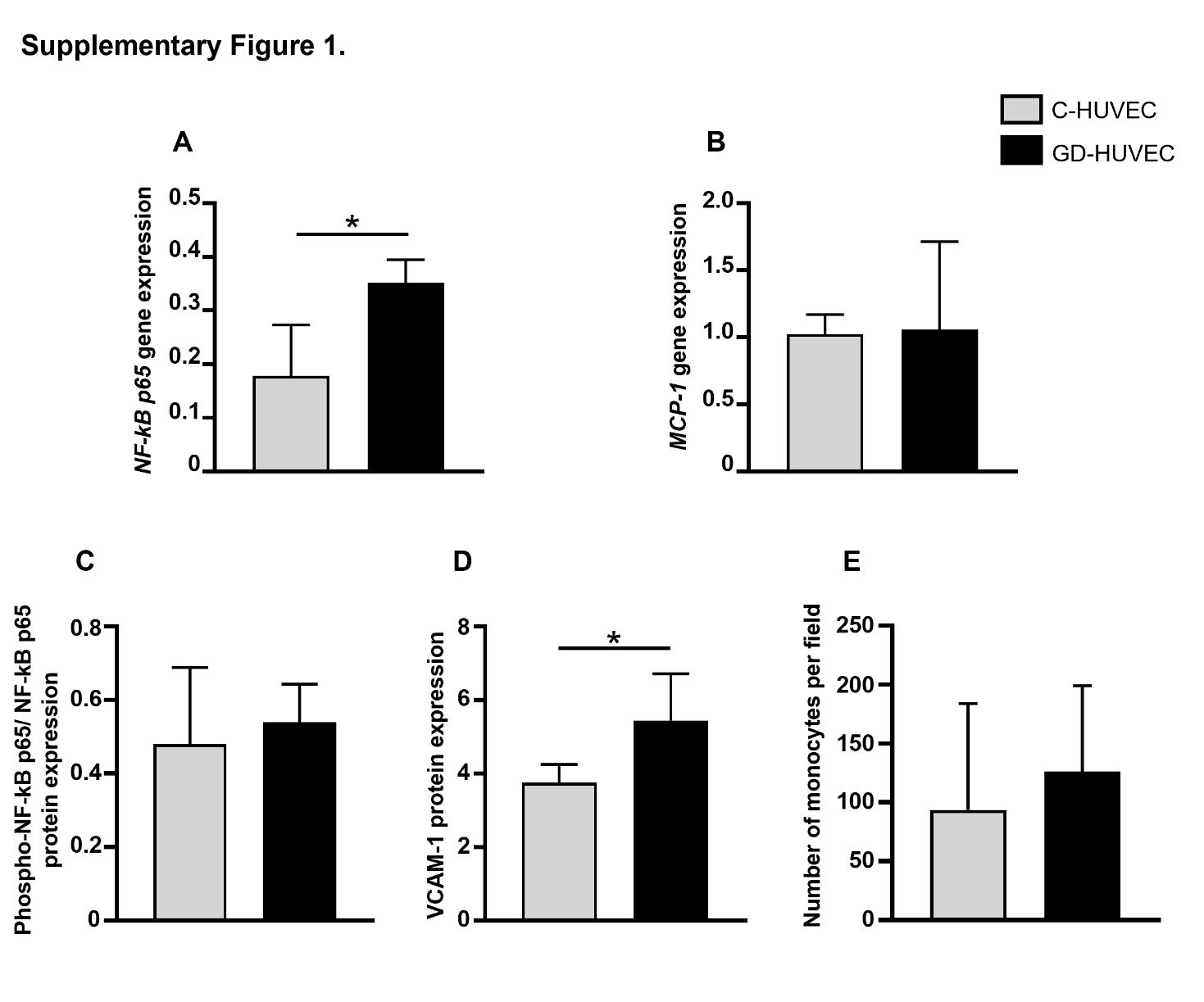
**

**Supplementary Figure 2.** Changlot Real and Picual phenolic extract treatment at different concentrations reduces VCAM-1 protein expression in both cell types. VCAM-1 protein levels in C- (A) and GD-HUVEC (B) after 24 hours treatment with Changlot Real and Picual phenolic extracts at different concentrations (0.1-50 μg/mL) in the presence and in the absence of 16 hours stimulation with TNFα (10ng/mL). Results are presented as the mean ± SD (n=4). Asterisks point out statistically significant differences between the selected conditions (*p<0.05;**p<0.01).

**
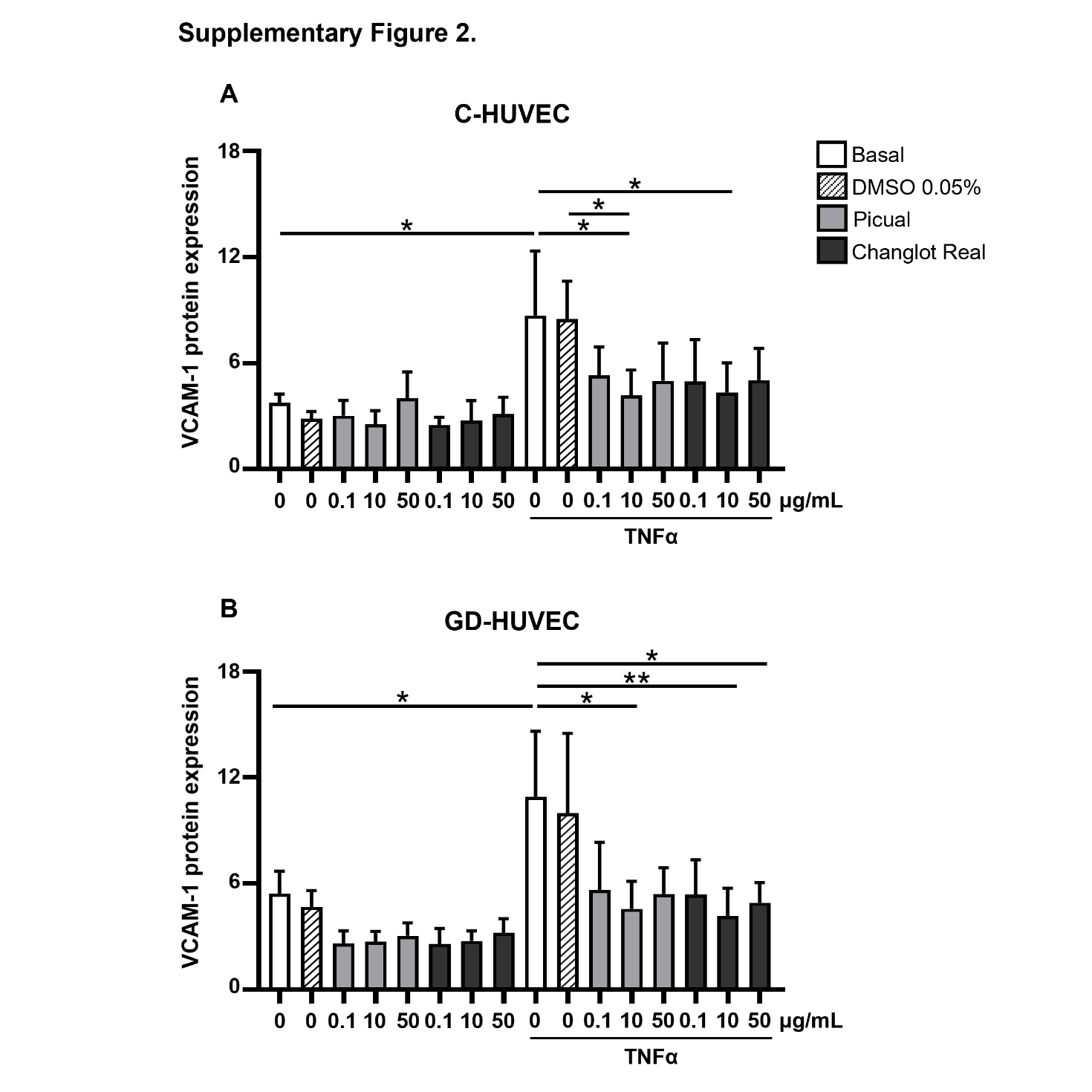
**

**
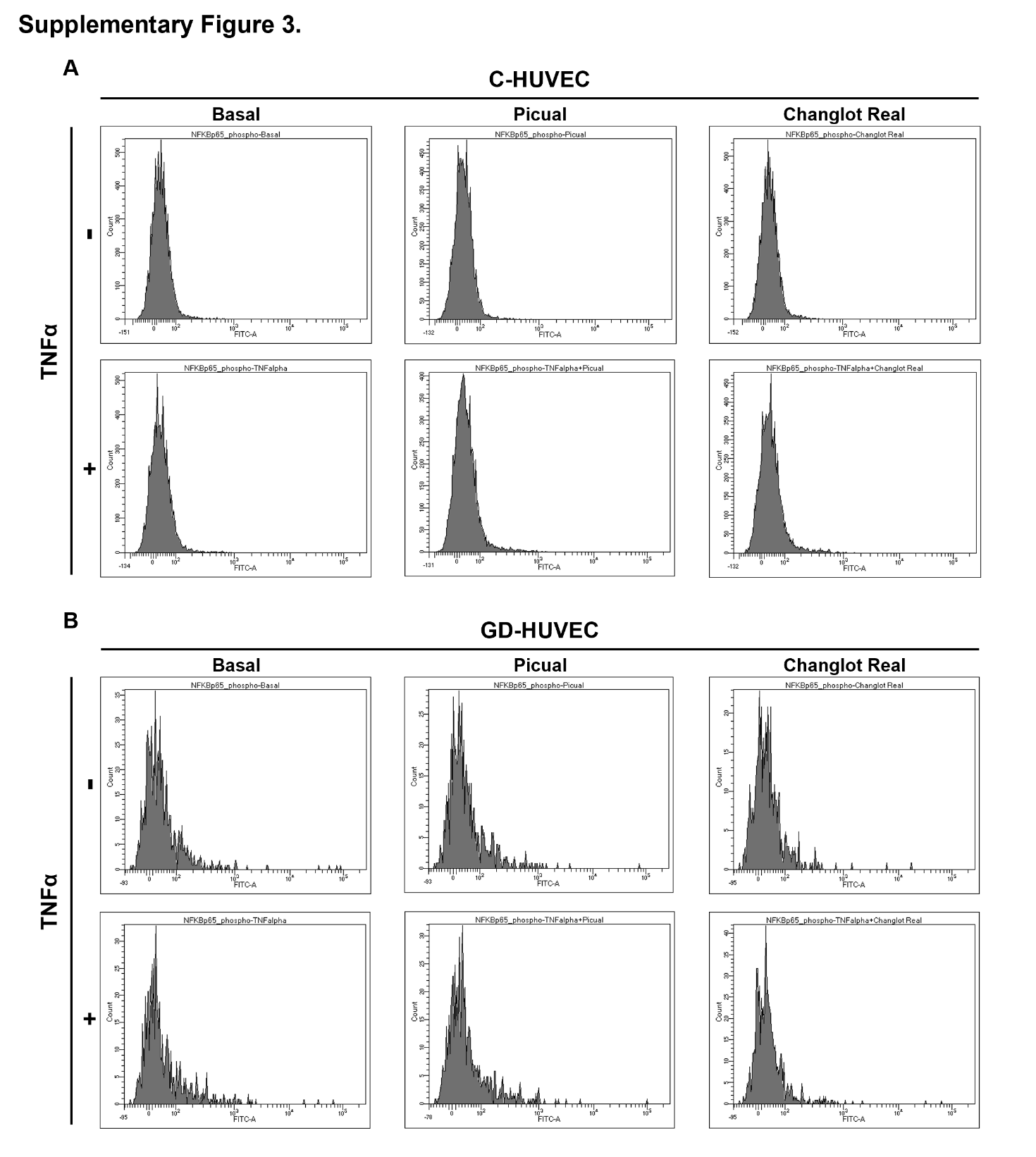
Supplementary Figure 3.** Representative flow cytometry graphs showing phosphorylated NF-κB p65 (Ser536) in C- (A) and GD-HUVEC (B) following 24-hour treatment with Changlot Real and Picual phenolic extracts (10 μg/mL), with or without 1-hour stimulation with TNFα (10 ng/mL).

**Supplementary Figure 4.** Representative flow cytometry graphs showing VCAM-1 protein expression in C- (A) and GD-HUVEC (B) following 24-hour treatment with Changlot Real and Picual phenolic extracts (10 μg/mL) with or without 16-hour stimulation with TNFα (10ng/mL).**
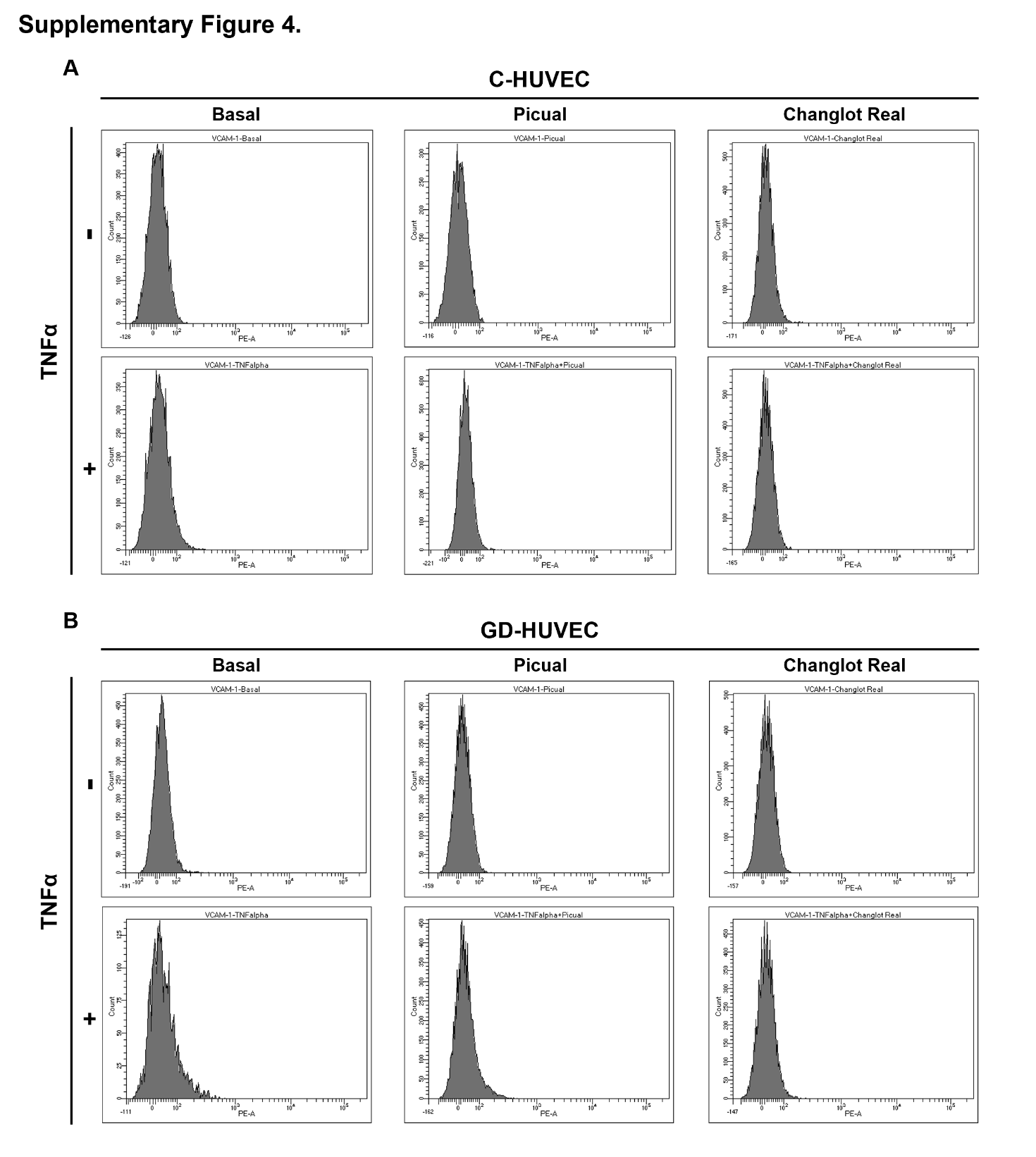
**
